# Editorial: Regulation of tumor immune surveillance: drug discovery and mechanisms

**DOI:** 10.3389/fimmu.2023.1352864

**Published:** 2024-01-05

**Authors:** Yuwei Zhang, Haofan Chen, Lingling Ou

**Affiliations:** ^1^ Operation Room, Affiliated Hospital of Beihua University, Jilin, Jilin, China; ^2^ Spine Surgery, The Sixth Affiliated Hospital of Sun Yat-sen University, Guangzhou, Guangdong, China; ^3^ School and Hospital of Stomatology, Guangzhou Medical University, Guangzhou, Guangdong, China

**Keywords:** cancer therapy, immune cell regulation, novel drugs, AlphaFold2, biopharmaceuticals

## Introduction

In recent years, there has been a surge in research on immune cell regulation in the field of cancer therapy, exploring diverse and captivating directions. From the activation of immune responses by outer membrane vesicles (OMVs) to overcoming the exhaustion of CD8+ T cells, these studies offer novel perspectives for advancing cancer treatment. Notably, research on the role of histone demethylases in gastric cancer regulation (Dong et al.) and the discovery of the anti-tumor effects of venom from red imported fire ants (Jeong et al.) provide opportunities for a better understanding of immunotherapy.

In terms of novel drugs and therapeutic strategies, the optimization of antibody structures predicted by AlphaFold2 has introduced entirely new possibilities for cancer treatment (Du and Huang). This series of studies not only expands our understanding of cancer biology but also innovatively contributes to the design of future therapeutic approaches.

Against this hopeful backdrop, research in the field of “Regulation of Tumor Immune Surveillance: Drug Discovery and Mechanisms” becomes particularly urgent. Delving into how immune cells regulate tumor monitoring and discovering new drugs and treatment mechanisms will open new chapters in cancer therapy, providing patients with more precise, efficient, and personalized treatment options.

## The role of immune cell regulation in cancer therapy

In recent years, the spotlight in cancer therapy has shifted towards the pivotal role of immune cell regulation. Research has illuminated a novel perspective on immune cell modulation, particularly through the activation of outer membrane vesicles (OMVs), leading to the significant expansion of Vγ9Vδ2 T cells (Firth et al.). This groundbreaking study unveiled, for the first time, the potent anti-tumor potential of Vγ9Vδ2 T cells upon OMV challenge, presenting promising directions for future therapeutic strategies. The integration of immune activation with the anti-tumor properties of Vγ9Vδ2 T cells offers innovative insights for the design of effective cancer immunotherapies.

Concurrently, intensive investigations have delved into the exhaustion state of CD8+ T cells within the tumor microenvironment, underscoring the urgency to overcome this condition (Guan et al.). CD8+ T cell exhaustion, a consequence of prolonged antigen stimulation, is particularly pronounced in the tumor microenvironment. Overcoming CD8+ T cell exhaustion has become a critical breakthrough for enhancing the effectiveness of immune therapy. Rapid advancements in various strategies, including immune checkpoint inhibition, transcription factor regulation, epigenetic modifications, metabolic modulation, and cytokine therapy, provide diverse tools to reverse the exhaustion state of CD8+ T cells.

Together, these two papers paint a comprehensive picture of the indispensable role of immune cells in cancer therapy. The study on OMVs introduces a pioneering concept for combating tumors by mobilizing specific immune cells, such as Vγ9Vδ2 T cells. Simultaneously, research on strategies to address CD8+ T cell exhaustion brings forth multiple possibilities for overcoming bottlenecks in immune therapy. The progressive advancements in this field are propelling the forefront of cancer immunotherapy, laying a robust foundation for the design of precise and efficient treatment modalities in the future. These developments not only capture the academic spotlight but also evoke significant interest in the pharmaceutical industry for innovative approaches to cancer treatment.

## The emerging potential of biopharmaceuticals in cancer treatment

Recent research suggests that biopharmaceuticals could represent a significant breakthrough in cancer therapy. Some striking discoveries indicate that venom from the red imported fire ant (RIFA) may harbor potent anti-tumor effects. Studies reveal that alkaloids, such as solenopsin A found in RIFA venom, exhibit notable inhibitory effects on tumors (Mo et al.).

The latest research demonstrates that RIFA venom selectively inhibits tumor cells by modulating the PI3K/Akt pathway. This discovery shares commonalities with other bio-toxins, including bee venom, snake venom, and toad venom. Noteworthy is the potential therapeutic effect of solenopsin A in RIFA venom, particularly showing significant inhibitory effects in psoriasis research.

This finding has ignited broad interest in the potential role of biopharmaceuticals in cancer treatment. Bio-toxins, such as bee venom and toad venom, have long been focal points in anti-tumor therapy research. They exert unique mechanisms against tumors, including the regulation of gene expression, promotion of apoptosis, and inhibition of tumor angiogenesis.

While current research on the mechanisms of RIFA venom in cancer treatment is still in its early stages, this discovery provides a fresh perspective for the development of novel cancer treatment drugs. Future research will delve deeper into the mechanisms of other components in RIFA venom, aiming to furnish more evidence for the innovation of biopharmaceuticals in cancer treatment. The ongoing progress in this field holds promise for delivering more effective and innovative treatment choices for cancer patients.

## Advancements in cancer therapy: innovations in novel drugs and treatment strategies

Remarkable progress is underway in the research and development of novel drugs and treatment strategies within the realm of cancer therapy. Recent studies underscore the pivotal regulatory role of histone demethylases in gastric cancer. In-depth investigations into key demethylases such as LSD1 and LSD2 offer new theoretical insights into the involvement of histone demethylation in the development and prognosis of gastric cancer (Dong et al.). On a different front, a therapeutic strategy targeting melanoma introduces the novel drug M-DM1. This drug selectively reduces M2-TAMs, resulting in the inhibition of tumor growth, migration, and invasion, showcasing promising anti-tumor effects and laying the foundation for future treatments (Jeong et al.). Additionally, research on trabectedin (TRB) and lurbinectedin (LUR) unveils their impact on immune metabolism, suggesting that these drugs can induce metabolic reprogramming to activate human macrophages, opening new possibilities for designing novel immune metabolic interventions (Povo-Retana et al.).

In the field of drug design, AlphaFold2’s predictions of molecular structures offer fresh insights into humanizing antibodies. Through structural optimization, studies have successfully designed humanized antibodies with enhanced anti-tumor effects, instilling new hope into cancer treatment (Du and Huang). This series of research highlights the continuous emergence of novel drugs and treatment strategies in cancer therapy, providing patients with more personalized and efficient treatment choices. These studies not only enrich our understanding of cancer biology but also chart the course for future clinical practices and drug development.

## Summary

In the current wave of research on immune cell regulation and novel drug treatment strategies, we are witnessing tremendous progress in the field of cancer therapy. From the activation of immune cells to addressing the exhaustion of CD8+ T cells in the tumor microenvironment, research continuously provides new perspectives and solutions for cancer treatment. Specifically, the study on outer membrane vesicles (OMVs) activating the immune system demonstrates a novel approach to combat tumors by mobilizing specific immune cells, such as Vγ9Vδ2 T cells. Simultaneously, strategies targeting the exhaustion of CD8+ T cells offer diverse tools to overcome bottlenecks in immunotherapy.

In drug development, the regulation of histone demethylases in gastric cancer and the introduction of novel drugs like M-DM1 show tremendous potential. The design of these therapeutic approaches emphasizes specificity, individualization, and efficacy, offering more precise treatment choices for patients. Moreover, the successful optimization of antibody structures predicted by AlphaFold2 opens new directions for the design of humanized antibodies, bringing renewed hope for the future of cancer therapy.

The emerging prospects of biopharmaceuticals, especially the in-depth exploration of potential anti-tumor components in the venom of red imported fire ants, inject new vitality into the field of cancer treatment. These biotoxins combat tumors through various mechanisms, providing ample space for the development of innovative and effective treatment modalities.

Looking ahead, we anticipate further advancements in cancer treatment through in-depth research into immune cell regulation mechanisms, optimized drug design, and the exploration of the potential of biopharmaceuticals. With the continuous progress of technology and deepening research, we are confident in providing patients with more personalized, precise, and efficient cancer treatment options, bringing more hope to the fight against cancer. The ongoing innovation in this field will be a highlight of future medical research, offering a beacon of hope for clinical practice and patients.

## Author contributions

LO: Writing – review & editing. YZ: Writing – original draft. HC: Writing – original draft.

